# Evaluating the Stability of PLA-Lignin Filament Produced by Bench-Top Extruder for Sustainable 3D Printing

**DOI:** 10.3390/ma16051793

**Published:** 2023-02-22

**Authors:** Siti Aisyah Syazwani Zaidi, Cham Eng Kwan, Denesh Mohan, Shuhaida Harun, Abdullah Amru Indera Luthfi, Mohd Shaiful Sajab

**Affiliations:** 1 Research Center for Sustainable Process Technology (CESPRO), Faculty of Engineering and Built Environment, Universiti Kebangsaan Malaysia, Bangi 43600, Selangor, Malaysia; 2 Department of Chemical and Process Engineering, Faculty of Engineering and Built Environment, Universiti Kebangsaan Malaysia, Bangi 43600, Selangor, Malaysia

**Keywords:** 3D printing filament, biocomposites, biodegradable, lignin

## Abstract

As additive manufacturing continues to evolve, there is ongoing discussion about ways to improve the layer-by-layer printing process and increase the mechanical strength of printed objects compared to those produced by traditional techniques such as injection molding. To achieve this, researchers are exploring ways of enhancing the interaction between the matrix and filler by introducing lignin in the 3D printing filament processing. In this work, research has been conducted on using biodegradable fillers of organosolv lignin, as a reinforcement for the filament layers in order to enhance interlayer adhesion by using a bench-top filament extruder. Briefly, it was found that organosolv lignin fillers have the potential to improve the properties of polylactic acid (PLA) filament for fused deposition modeling (FDM) 3D printing. By incorporating different formulations of lignin with PLA, it was found that using 3 to 5% lignin in the filament leads to an improvement in the Young’s modulus and interlayer adhesion in 3D printing. However, an increment of up to 10% also results in a decrease in the composite tensile strength due to the lack of bonding between the lignin and PLA and the limited mixing capability of the small extruder.

## 1. Introduction

3D printing, also known as additive manufacturing, is a type of digital fabrication technology. It creates physical objects by layering materials according to a digital design or geometric representation [1]. Scientists have developed and studied various 3D printing technologies, with different techniques currently available on the market. Fused deposition modeling (FDM) 3D printing has become a cost-effective technology with the availability of commercial 3D printers and filaments at reasonable prices. However, this new technology is also associated with a significant amount of waste, mainly due to a lack of technical skills and issues with the quality of some 3D printers. This waste is primarily generated from support materials, failed products, and broken plastic parts [2,3,4].

Another of the ongoing issues in 3D printing is the structure of spaces or cavities between layers, which can affect the mechanical strength of printed objects when compared to those produced using traditional injection-molding processes. To address this issue, researchers are investigating the use of sustainable additives or fillers in the printing process to enhance layer adhesion and tensile properties when 3D printing at complex angles [5].

In Malaysia, the oil palm empty fruit bunch (OPEFB) is one of the major biomasses produced from palm oil mills and comprises cellulose, hemicellulose and lignin; it can be investigated extensively to increase polymer biodegradability, mechanical characteristics and minimize the burden of the fossil fuel business [6]. Among the components in the lignocellulose, cellulose is isolated from the lignocellulosic biomass and is widely utilized in many applications. Meanwhile, lignin is the by-product of the process and is considered as one of the main waste materials produced in comparatively large amounts which show great economic potential for a variety of high-value bio-based products [7]. Additionally, lignin is a highly branched phenolic polymer that makes up 15 to 30% of the lignocellulose biomass by weight and binds well compared to cellulose due to its hydrophobic properties [8,9,10]. Therefore, lignin is being extensively studied with the aim of replacing inorganic fillers to enhance the mechanical properties of the polymer.

Lignin is a highly versatile material for sustainable development and reducing the carbon footprint. It can be extracted using various techniques that result in different structures and molecular weights, making it attractive for the production of bio-based products. Lignin is gaining attention worldwide due to its low cost, renewability, biodegradability, and high carbon content, especially for 3D printed materials [11,12,13]. Incorporating micro-sized lignin into FDM 3D printing filaments can enhance the mechanical properties of the printed products, making them more environmentally friendly and biodegradable.

The addition of lignin to composites replicates the natural conditions of the plant cell wall and improves the interfacial adhesion of the lignocellulosic matrix, resulting in enhanced mechanical properties such as Young’s modulus, tensile strength, flexural strength, and improved wettability [14,15,16,17,18]. The incorporation of lignin additionally increases the maximum load before fracture, making the resulting materials suitable for various healthcare applications, including wound healing. However, using neat lignin as a filament is not feasible due to its high heat transition temperature and resistance to flow [13,19]. To improve the mechanical properties of biocomposites, extensive research has been conducted on lignin modification and after-treatment methods to achieve greater compatibility between fibers and the polymer matrix [20,21,22,23].

Therefore, this work aimed to study the stability and compatibility of in-house processing filament via a bench-top extruder at different formulations of polylactic acid (PLA) and extracted lignin. Organosolv lignin, extracted from the OPEFB, was blended with various ratios of PLA pellets. These composite pellets were extruded with a filament diameter of about 1.75 mm and 3D-printed using FDM, to assess the improvement in mechanical properties of the composite over neat PLA. Detailed chemical and thermal analysis was conducted to examine the chemical interactions and structure of the composite, and their relationship to the mechanical properties of the 3D-printed parts.

## 2. Materials and Methods

### 2.1. Materials

The isolation of lignin from OPEFB fibers (particle sizes of 106 to 500 µm, Szetech Engineering Sdn. Bhd, Selangor, Malaysia) was carried out using 90% formic acid (FA) (Merck, Darmstadt, Germany). PLA was received in natural pellets (Ingeo biopolymer 3052, Plymouth, MN, USA). 

### 2.2. Preparation of Composites

Organosolv extraction of the lignin process was carried out according to previously described procedures [24,25]. Briefly, FA at a ratio of 1:30 was reacted to OPEFB fibers (10 g of OPEFB fibers to 300 mL of 90% FA) and stirred at 600 rpm at 95 °C at a constant heat for 2 h. Separation of the pulp and the extracted organosolv lignin was conducted using a vacuum filter (MVP 10, IKA, Staufen, Germany). The organosolv lignin and FA was then recovered through a rotary evaporator (RE 600, Yamato Scientific Co., Ltd., Tokyo, Japan). The final product was rinsed out multiple times to remove excess FA and dried overnight in an oven before being stored in a desiccator.

In the preparation of filament composites, the organosolv lignin was used as a filler for PLA and was blended with 3–15% of lignin ratio compositions. The mixture composition of biopolymer formulation was mixed using a mechanical stirrer (IKA RW20, Staufen, Germany) at 2000 rpm for 30 min. The samples were kept in a convection oven at 50 °C for 6 h with the moisture content <0.5% before the filament extrusion.

### 2.3. Filament Extrusion

The filament extrusion was carried out using a 3devo Composer 350 bench-top extruder and a single mixing screw extruder with a nozzle diameter of 4 mm (3devo, Utrecht, The Netherlands). Prior to the filament extrusion, the extruder was preheated and cleaned using virgin high-density polyethylene (HDPE) to remove the excess materials. The extruder was preheated up to 230 °C until the temperature suited the composite formulation. Gradually, a total of 100 g of each formulated samples was deposited onto the extruder hopper. The control parameter for the filament extrusion was designed to create filament thickness in the diameter ±1.75 mm. The extrusion profiles were set at four different heating zones (165, 180, 180 and 170 °C), 80% of cooling fan speed and an extrusion speed of 3.5 rpm, where the lower extrusion speed helps to reduce the amount of fluctuation in the diameter of the filament. The extruded filament composites were spooled and stored in the desiccator until further use. The extruder was cleaned once again using HDPE before the other compositions were repeated. All samples were purged until the flow of extrusion was consistent. The filament diameter, extruder speed and temperatures of all samples were real-time monitored using DevoVision (3devo, Utrecht, The Netherlands).

The extruded filaments were analyzed with a commercial FDM 3D printer with a 0.4 mm nozzle (Prusa i3 MK3S Prusa Research, Prague, Czech Republic). The CAD model (.stl file) utilized in this study was an ASTM D638 type V standard tensile specimen for tensile property analysis. The file was sliced to a G-code file through slicer software (PrusaSlicer, Prague, Czech Republic). The printing profiles were varied by the nozzle temperature, infill, layer height, extrusion speed, and printing speed for the best printing structure (layer height: 0.30 mm; infill percentages: 15%; infill pattern: rectilinear at 45° lines; print speed: 60 mm/s). In order to determine the interlayer adhesion of infused lignin, the printing orientation was set at 0 and 90° on top of the build plate (horizontal and vertical printed layers).

### 2.4. Characterization

Chemical characteristics of synthesized filament composites were determined using attenuated total reflectance Fourier transform infrared, ATR-FTIR (ALPHA FTIR Spectrometer, Bruker, Billerica, MA, USA) at a resolution of 1 cm^−1^ in the range of 4000 cm^−1^ to 500 cm^−1^. Thermochemical analyses were determined using a differential scanning calorimeter (DSC-50, Shimadzu Corporation, Kyoto, Japan) under nitrogen circumstances from 25 to 250 °C at a heating rate of 10 °C/min. In addition, the thermogravimetric analysis (TGA) was performed by changes in the thermal degradation of composite samples at temperatures ranging from 25 to 600 °C under nitrogen circumstances at a heating rate of 10 °C/min.

The mechanical characterization of the 3D printed samples was measured using Instron^®^ Electromechanical Universal Testing Systems 3300 Series at 10 mm/min with a load cell of 1 kN. All the data reported were based on the mean of five replicates (*n* = 5). The morphological arrangement of layer-by-layer adhesion was investigated using a field emission scanning electron microscope (FESEM) (Merlin Compact, Zeiss Pvt LtD., Oberkochen, Germany). The cross-sectional samples were sputter-coated with platinum before viewing to reduce the charging effect of the samples.

## 3. Results

### 3.1. Chemical and Thermochemical Characterizations

**Figure 1:**
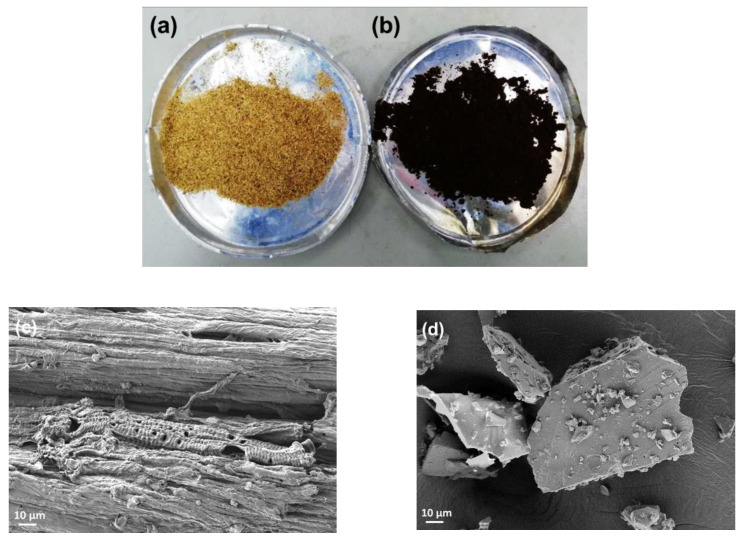
Structure of (**a**) OPEFB fibers (**b**) extracted lignin and the micrograph of (**c**) OPEFB after extraction (**d**) organosolv lignin.

Organosolv lignin that underwent extraction from the OPEFB was processed using an organosolv method that utilizes 90% FA and a rotary evaporator to isolate the organosolv lignin from the FA. After the extraction and separation process, OPEFB fibers became more brownish, as shown in Figure 1. Because the OPEFB was still in particle sizes of 106–500 µm, the blending process in the bench-top extruder could damage the extruder screw in the filament making [24]. The morphological structure of the OPEFB after extraction (see Figure 1c) shows the fibrils of cellulose after the dissolved lignin has been extracted with the FA. While the dark brown of the isolated lignin subsequently dehydrated, yield at 22.5% from the neat OPEFB fibers shows the uneven structures on the micrograph image of FESEM in Figure 1d.

**Figure 2:**
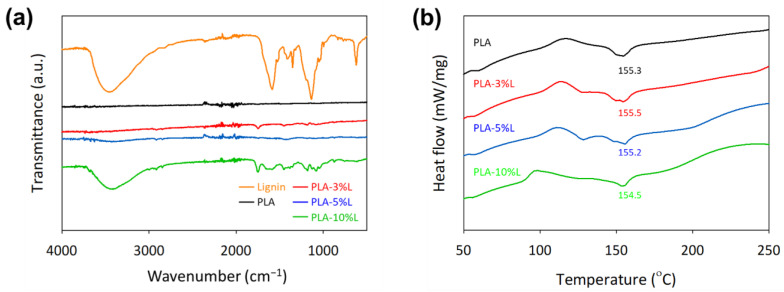

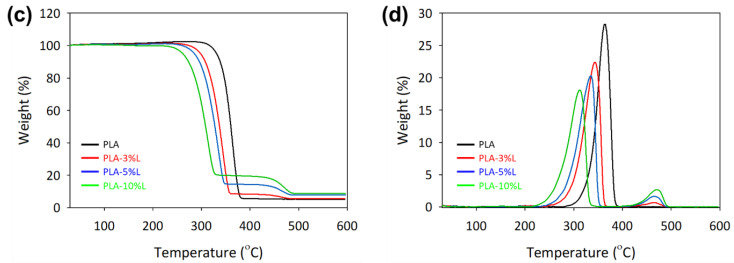
Chemical characterization by (**a**) FTIR and thermogram analysis of (**b**) DSC (**c**) TGA weight loss and (**d**) first derivative TGA analyses of extruded filament composites at different lignin compositions.

The presence of functional groups of isolated organosolv lignin was; the blended filament composites are shown in Figure 2a. The peaks band can be highlighted between 3400 and 3600 cm^−1^ and indicate the presence of a hydroxyl group, O–H, whereas the band at 2940 cm^−1^ indicates the presence of a methyl group, C–H, whereas the bands at 1716 cm^−1^ and 1500 to 1600 cm^−1^ indicate the presence of a carbonyl group, C=O, and an aromatic group, C=C. Following this, 1410 to 1470 cm^−1^ show an asymmetrically deformed C–H group in –CH_3_ and –CH_2_, while 1350 cm^−1^, 1212 cm^−1^, 1130 cm^−1^ to 1110 cm^−1^ explain the S group of the C–O stretch, phenolic OH and ether in S and G, as well as the S group of the C–H stretch [2,25]. Despite the increment in lignin as a filler, the spectrum shows no substantial differences and thus demonstrates that the chemical structure of the filament composites does not change during process extrusion.

The differential scanning calorimetry transitions depicted in Figure 2b indicate that the glass transition temperature, T_g_ and melting temperature, T_m_ for PLA were recorded at 54 and 155 °C, respectively. The previous work based on organosolv extraction lignin from OPEFB fibers exhibited a thermoplastic characteristic with a glass transition temperature, T_g_ around 97 °C [24]; thus, there was a slight increment in the glass transition value from 120 to 130 °C with the increment in lignin in the filament composites. The glass transition temperature was still at 50 to 60 °C. 

The thermal decomposition of filament composites was clearly disrupted by the addition of lignin as a filler (see Figure 2c,d). The PLA–lignin composites exhibit a two-step degradation process. The first step is due to the thermal degradation of the PLA polymer chain, which is associated with active pyrolysis and oxidation, and occurs within a temperature range of 150 to 400 °C [26]. The second step is associated with the lignin decomposition, which produces volatile gases and a relatively high char carbon residue, and occurs slowly at temperatures above 450 °C [24]. The presence of the char carbon residue in the filament composites results in a higher level of decomposition, which increases with the higher lignin content, reaching up to 18.54% at 600 °C, as shown in Table 1. The higher residual weight of 39.71% at 600 °C is due to the breaking of the bonds of the lignin molecules, which subsequently release phenols in vapor phases [24]. In contrast, only one degradation step was observed for the neat PLA, consistent with previous reports [18,27]. Although, theoretically, PLA polymers and filament composites have a higher glass transition temperature than organosolv lignin, nevertheless their glass transition temperatures are nearly equivalent.

All filament composites completely disintegrate at temperatures of up to 600 °C and this is coupled with a volatile PLA product that decomposes rapidly between 250 and 390 °C. Despite the fact that the composites gradually deteriorated at temperatures above 250 °C, this would not have a major impact on the polymer degradation during the composite extrusion process. In this work, the established temperatures that were used in the filament extrusion and FDM 3D printing were below the identified deteriorating temperature; both procedures had temperature settings of 165/180/180/170 °C and 215 °C, respectively.

### 3.2. Stability of Filament Composites

**Figure 3:**
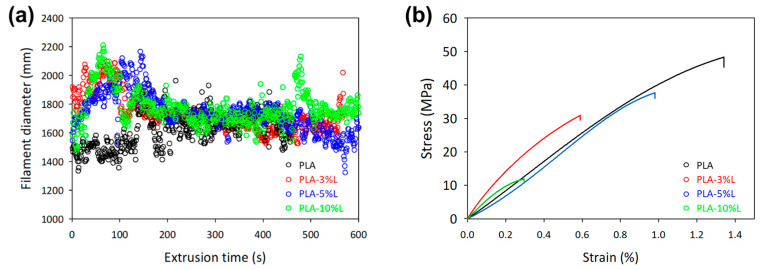
The effect of lignin composition on the stability of filament extrusion at (**a**) filament diameter and (**b**) mechanical strength.

The filament diameter over extrusion time was provided in real-time monitoring software, DevoVision (see Figure 3a). Although each composites formulation was successful in achieving a desired filament diameter range around 1.75 mm, nevertheless the impacts of lignin composition on extrusion stability were shown at the initial extrusion time. Maintaining the filament diameter within the prescribed range takes time and was reasonably challenging on PLA-10%L, as it required more time to achieve the desired filament diameter. Although the mixture composition was mixed vigorously using a mechanical stirrer, lignin agglomerates and uneven distribution are a typical occurrence during extrusion and high heat [25,28,29,30]. As a result, the filament diameter started to indicate a fluctuating trend at a higher lignin composition.

The inconsistent distribution of lignin during the filament extrusion may as well be observed at its mechanical analysis in Figure 3b and summarized in Table 2. The highest tensile strain was achieved by PLA at 49.57 MPa. While adding lignin decreased the strength slightly to 37.33 MPa with a 3–5% composition, a drastic reduction to 12.8 MPa was observed at a 10% lignin composition. This decline in mechanical strength can be attributed to the unequal distribution of lignin at higher ratios, likely due to the limited mixing capability of the bench-top filament extruder [19]. However, the Young’s modulus of the lignin-incorporated samples showed an improvement of up to 419 MPa, an 11% increase from neat PLA.

### 3.3. Interlayer Adhesion of Infused Lignin

**Figure 4:**
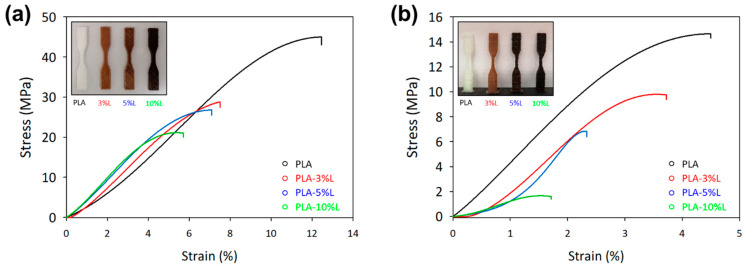
Mechanical strength of the 3D printed product of neat biopolymer and biopolymer composites at different (**a**) horizontal (0°) and (**b**) vertical (90°) orientations. Subset images show the 3D printed product as a comparison.

All printing parameters were set as the same for the two types of different orientations, horizontal (0°) and vertical (90°) (see Figure 4). Further to that, the nozzle and build plate temperatures were adjusted according to the temperature profiles, which was 215 °C for the PLA material and 60 °C for all other materials. The tensile strength analysis on printed samples according to the ASTM D638 type V standard specimens is depicted in Figure 4 and the data are summarized in Table 2.

The decreasing trend in tensile strength for composites is most likely due to lignin’s lower tensile strength when compared to PLA. In general, the tensile strength is influenced by lignin–lignin intermolecular interactions, PLA–PLA interactions, and PLA–lignin interactions, as well as the rigidity of lignin particles [31]. Furthermore, this trend is attributed to a lack of interfacial bonding between constituents, particularly the hydroxyl groups found in unmodified lignin macromolecules. Increased miscibility can thereby be achieved by replacing the hydroxyl group with other functional groups that can strengthen the bond between the matrix and the filler [20]. Similar results are summarized in Table 3 and while most cases showed reduced tensile strength, the use of lignin as a biofiller greatly benefits the 3D printing filament functionalization. Although lignin incorporation into the biocomposite reduces the modulus of the tensile strength, these mechanical properties can be improved by optimizing 3D printing temperatures [32].

Therefore, 3D printing at a horizontal angle of 0°and 90°, PLA-3%L (0°) shows the highest Young’s modulus among other sample formulations. This emphasizes the importance of including lignin in polymer formulations, as both the orientation set-ups show the improvement in Young’s modulus compared with the neat PLA (34 and 20% increment of 0°and 90° with the addition of 3 and 5% of lignin, respectively). Therefore, the improvement in Young’s modulus is mainly attributed to the excellent role of lignin as a rigid filler that increased stiffness. This increase in stiffness can be linked to both hydrophobic interactions and hydrogen-bond electrostatic forces between the lignin and the polymer [33]. In Table 2, it can be observed that the Young’s modulus of PLA lignin composites gradually increases, but then begins to decrease as higher amounts of lignin are incorporated. This decrease in the modulus with the higher lignin content is a typical occurrence in green composites, as the increased presence of filler in the matrix reduces the molecular mobility of the polymer chains, as referenced in [34].

The increment in Young’s modulus is comparable from the previous findings associated with the improvement in the interlayer adhesion of 3D printing [13,19,24,35]. Additionally, the mechanical analysis of 3D printed objects with lignin showed a higher tensile strength and modulus compared to those printed with neat PLA (please refer to Table 2). The inclusion of lignin improved the interlayer adhesion, resulting in a 57% and 31% increase in the Young’s modulus of PLA-3% and PLA-10%, respectively. Although a higher lignin content was found to have a lower impact, the printing process nevertheless managed to improve tensile and modulus properties by 82% with the PLA-10%L blend.

**Figure 5:**
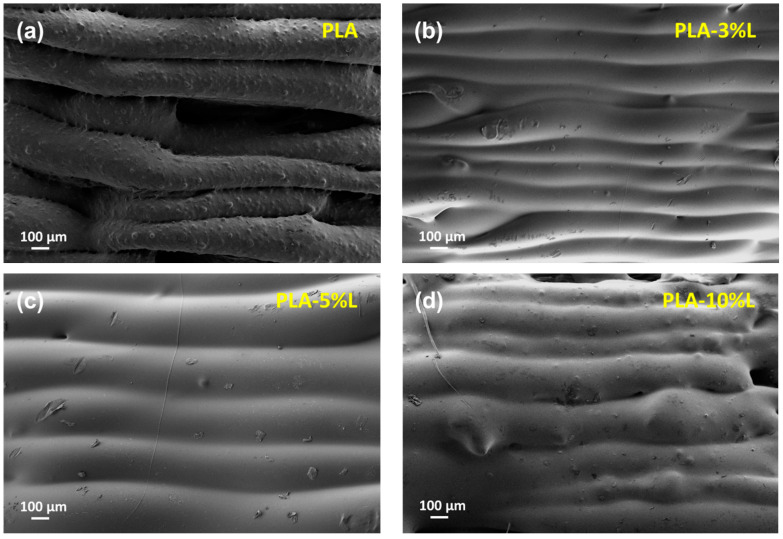
Cross-sectional area micrograph of 3D printed (**a**) neat PLA (**b**) PLA-3%L (**c**) PLA-5%L and (**d**) PLA-10%L.

**Figure 6:**
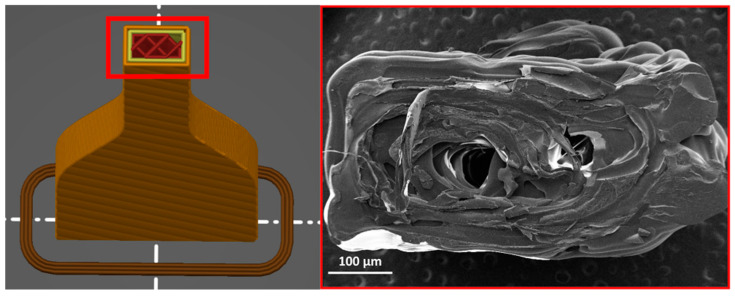
Interlayer adhesion of lignin incorporated of PLA-3%L at printing orientation of 90°.

Fractured tensile samples underwent mechanical testing and were then examined for their morphology to study the interaction of lignin with the polymer matrix (see Figure 5). Optimal printing parameters showed that the manually extruded PLA matrix had noticeable layers and provided significant gaps throughout the printing process. However, the incorporation of lignin from 3 to 10% led to proper blending between the layers. As a result of a higher lignin content and poor distribution process in the small extruder system, the morphological structure of PLA-10%L revealed more lignin agglomeration in the polymer matrix, allowing for weaker mechanical properties compared to other lignin formulation ratios. As shown in Figure 6, the interlayer adhesion of lignin-incorporated PLA at a 3% concentration resulted in a disrupted infill structure when compared to the slicer program. The fracture of the ASTM D638 type V model at 90° demonstrated no delamination between the layers of print, but rather a rugged fracture between the printed layers. 

**Table 3:** Previous research on using lignin as a biofiller in FDM filament production.

Polymer/ Composite	Lignin (wt.%)	Filament Extrusion Technique	Printing Temp. (°C)	Tensile Strength (MPa)	Improvement	Ref.
PLA	40	Single-screw	215	45.65	50% antioxidant potential/cm^2^	[11]
PHB	50	Twin-screw	190	-	34–78% less warpage	[36]
ABS/NBR/ carbon fiber	40	Single-screw	230	65	Improved interlayer adhesion	[13]
PLA	3	Single-screw	185–205	-	Antioxidant property	[19]
PLA/PEG	20	Twin-screw	210	50.84	Improved strength and elongation	[37]

### 3.4. Forecasting the Mechanical Strength

The mechanical strength data of 3D printed samples was further validated with the integration of an artificial intelligence hybrid technique called the adaptive neuro fuzzy inference system (ANFIS). The study aimed to predict the tensile strength of the printed samples under different conditions using lignin concentration (3, 5 and 10 wt%), infill (15 and 30%), and orientation (0° and 90°) of the printed samples as input variables. The tensile strength was obtained through UTM testing, and the ANFIS model was then trained and tested using a Sugeno-type fuzzy inference system [38].

**Figure 7:**
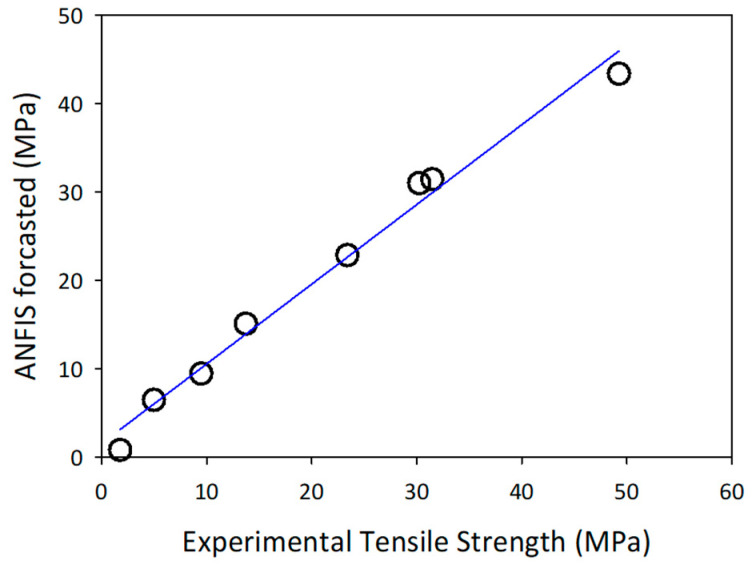
Linear regression between the predicted values versus experimental tensile strength values during testing using the ANFIS model.

In this study, the ANFIS model structure consisted of multiple rules, with 27 rules generated by tuning the three process parameters (lignin concentration, infill, orientation). The training data were loaded into the network and then used to train and test the fuzzy inference structure by adjusting the membership function parameters for optimal performance. The output function was constructed linearly with a single response. The best prediction for optimum tensile strength was achieved using 3 wt.% lignin at 15% infill and horizontal orientation (0°), with the highest strength being achieved by neat PLA at 15% infill and horizontal orientation. The ANFIS model results were consistent with the experimental results, as shown in Table 4. The predictive model for estimating the tensile strength of the FDM fabricated parts appears to be highly reliable, as indicated by the high coefficient of determination (*r*^2^ = 0.986), as shown in Figure 7. This highlights the effectiveness of the developed model and set-up as reported by other researchers [39,40,41].

## 4. Conclusions

3D printing filament made from a combination of biopolymer PLA and biofiller lignin was extruded using a bench-top filament maker with an optimal diameter of 1.75 mm. All factors such as the extrusion temperature, speed, and cooling fan speed were carefully controlled and recorded, with the best results being achieved at 165/180/180/170 °C for the biocomposite. The study found that using a 3% lignin formulation resulted in the highest Young’s modulus for the biocomposite filament. The ANFIS model was used to predict and validate the results and it was found that the biocomposite has potential for use in 3D printing even in a small-scale production.

## Figures and Tables

**Table 1:** Glass transition temperature (T_g_), sample residue percentages, and peak maximum decomposition rate filament composites.

Sample	T_g_ (°C)	Residue at 600 °C (%)	DTG_max_ (°C)
PLA	155.3	5.17	364.89
PLA-3%L	155.5	5.54	344.64
PLA-5%L	155.2	7.84	335.87
PLA-10%L	154.5	18.54	295.81

**Table 2:** Mechanical properties of extruded filaments and 3D printed PLA and PLA incorporated with lignin at different horizontal (0°) and vertical (90°) orientations.

Sample	Tensile Strength (MPa)	Tensile Strain (%)	Young’s Modulus (MPa)
Filament PLA	49.57 ± 0.89	38.66 ± 0.50	377 ± 0.03
Filament PLA-3%L	31.01 ± 0.93	18.39 ± 0.64	417 ± 0.02
Filament PLA-5%L	37.33 ± 0.75	24.41 ± 0.83	419 ± 0.04
Filament PLA-10%L	12.8 ± 0.57	12.54 ± 0.22	288 ± 0.05
PLA (0°)	49.25 ± 0.21	12.85 ± 0.16	488 ± 0.03
PLA-3%L (0°)	31.48 ± 0.30	7.70 ± 0.20	654 ± 0.02
PLA-5%L (0°)	30.23 ± 0.12	7.37 ± 0.13	548 ± 0.04
PLA-10%L (0°)	23.38 ± 0.09	5.8 ± 0.24	524 ± 0.05
PLA (90°)	13.72 ± 0.10	4.47 ± 0.05	479 ± 0.04
PLA-3%L (90°)	9.48 ± 0.15	3.85 ± 0.03	489 ± 0.02
PLA-5%L (90°)	4.96 ± 0.08	2.84 ± 0.04	578 ± 0.03
PLA-10%L (90°)	1.75 ± 0.12	1.89 ± 0.07	209 ± 0.05

**Table 4:** Comparison experiment data with forecast data ANFIS.

Sample	Tensile Strength (MPa)	ANFIS Forecast (MPa)
PLA (0°)	49.25 ± 0.21	43.40
PLA-3%L (0°)	31.48 ± 0.30	31.48
PLA-5%L (0°)	30.23 ± 0.12	31.04
PLA-10%L (0°)	23.38 ± 0.09	22.89
PLA (90°)	13.72 ± 0.10	15.11
PLA-3%L (90°)	9.48 ± 0.15	9.48
PLA-5%L (90°)	4.96 ± 0.08	6.50
PLA-10%L (90°)	1.75 ± 0.12	0.82
